# A Case of Bilateral Retinal Vasculitis in Atypical Cogan Syndrome

**DOI:** 10.7759/cureus.66984

**Published:** 2024-08-16

**Authors:** Chie Inokuchi, Shigeru Sato, Makoto Terada, Sato Uematsu, Setsuko Shirai

**Affiliations:** 1 Ophthalmology, Itami City Hospital, Itami, JPN; 2 Ophthalmology, Osaka University, Osaka, JPN; 3 Immunology and Allergy, Itami City Hospital, Itami, JPN

**Keywords:** multiple erythema, auditory disturbance, retinal neovascularization, uveitis, retinal vasculitis, cogan syndrome

## Abstract

Cogan syndrome (CS) is a rare chronic inflammatory disease characterized by ocular and inner ear inflammation. Well-known ocular manifestations include non-syphilitic interstitial keratitis (IK); however, some cases are not associated with IK. Inner ear symptoms include sensorineural hearing loss, rotatory vertigo, and tinnitus, which can become irreversible without timely treatment. Therefore, early and appropriate diagnosis and therapeutic intervention are important. However, due to its rarity, few physicians have encountered CS and early diagnosis is difficult. In this report, we present the details of the diagnosis and treatment of an atypical CS. The patient was a 44-year-old Japanese woman who was admitted to the Department of Immunology and Allergy at Itami City Hospital (Itami City, Hyogo, Japan) due to a persistent fever of approximately 40°C for nine days. Multiple erythematous lesions appeared on both lower legs, and she experienced decreased vision in her left eye. Uveitis with retinal vasculitis was observed in both eyes and the optic nerve head showed remarkable swelling in the left eye. Hearing tests revealed impaired hearing in both ears. Based on these findings, we diagnosed atypical CS and initiated systemic and topical steroid therapy. Approximately two weeks later, visual acuity and hearing levels improved. Fluorescein angiography (FA) revealed a non-perfusion area in both eyes, and retinal photocoagulation was performed using a pattern-scanning laser. Eighteen months after the laser irradiation, retinal neovascularization (RNV) was observed in the area where the laser was applied to the left eye; therefore, an additional laser was applied. Combination therapy with steroids and immunosuppressive drugs was continued until the patient’s last visit three years later and she did not experience any recurrence of uveitis or hearing loss. In this case, a pattern-scanning laser was used for retinal photocoagulation to prevent RNV; however, RNV occurred within the area of the laser spots. In such cases of retinal capillary occlusion due to vasculitis, it may be better to close the spacing or use a conventional laser system. In the presence of retinal vasculitis with systemic inflammation, CS should be suspected, and a hearing test should be performed, even in the absence of subjective symptoms. Early treatment and prevention of irreversible hearing loss should be necessary. Careful follow-up in collaboration with other departments is important for CS cases.

## Introduction

Cogan's syndrome (CS) was first described by Cogan in 1945 as a non-syphilitic interstitial keratitis (IK) with vestibular auditory symptoms such as rotatory vertigo, tinnitus, and hearing loss [1]. In 1980, Haynes et al. found that some patients with eye and ear symptoms similar to CS had vasculitis by reviewing 13 cases and sporadic cases reported over the last 20 years and concluded that CS was associated with inflammatory vasculitis in the central nervous system [2]. Consequently, they proposed dividing CS into classic and atypical types [2]. According to his report, classical CS presents with three symptoms: IK, vestibular dysfunction, and sensorineural hearing loss, as reported by Cogan [1,2]. Cases of vasculitis of large vessels, especially the aorta, have been classified as atypical [2,3]. However, in many cases, it is difficult to distinguish between classic and atypical CS because some patients do not have IK at the onset of the disease, whereas others develop IK over several years [4]. Systemic symptoms, such as vasculitis, are observed in approximately 30-50% of cases of atypical CS [5,6]. In this report, we present a case of atypical CS with bilateral occlusive retinal vasculitis and a detailed clinical course.

## Case presentation

**Figure 1:**
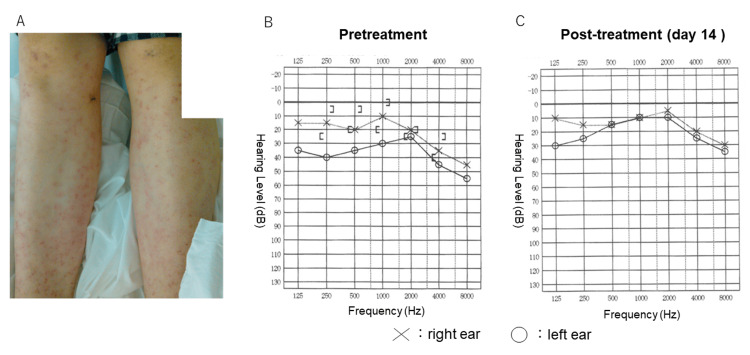
Photograph of multiple erythema and hearing test charts (A) Multiple erythematous lesions were observed on both lower legs. (B, C) The audiogram of pretreatment (B), and two weeks after treatment (C) symbols denote the following: X right ear, O: left ear

A 44-year-old Japanese woman with no medical and drug ingestion history visited her family doctor because she had a fever of approximately 40 °C for three days. Soon after, she developed multiple systemic erythematous lesions and decreased vision in her left eye. Nine days after onset, when she visited a former ophthalmologist, her corrected visual acuity of the decimal unit (CVA) in her left eye was 0.06. According to the referral letter, slit-lamp microscopic examination revealed inflammatory cells in the anterior chamber, and fundoscopy revealed retinal vasculitis with retinal hemorrhage in both eyes and optic nerve head swelling in the left eye. Her home doctor suspected Behçet's disease due to systemic multiple erythema and referred her to the Department of Immunology and Allergy at Itami City Hospital (Itami City, Hyogo, Japan) for detailed examination and treatment. At the first visit to our hospital, her body temperature was 36.6 °C, and her blood pressure was 92/41 mmHg in her bilateral arms. Physical examination revealed lymphadenopathy on the right side of the neck and multiple erythematous lesions in both lower legs (Figure 1A).

She had no oral and/or vulvar ulcers or vestibular cochlear symptoms such as hearing loss, tinnitus, or dizziness. Blood tests showed elevated C reactive protein (0.57 mg/dL, 0-0.3) and percentage of neutrophils (75.9%, 34.0-71.0). There was no significant elevation in autoantibodies, and none of the infections, including syphilis, showed elevated antibody titers (Table 1).

**Table 1:** Lab data CRP: C-reactive protein; RF: rheumatoid factor; ACE: angiotensin-converting enzyme; sIL-2: soluble interleukin-2 receptor; ANA: antinuclear antibody; SS-A: Anti-SS-A antibody; SS-B: anti-SS-B antibody; PR3-ANCA: proteinase 3 anti-neutrophil cytoplasmic antibody; MPO-ANCA: myeloperoxidase-anti-neutrophil cytoplasmic antibodies; PRP: rapid plasma reagin test; TpAb: treponema pallidum antibody

	Test result	Normal range		Unit
Leukocytes	43	37	to 87	10^2/L
Neutrophil	75.9	34	to 71.0	%
Eosinophil	0	0	to 8.0	%
Monocyte	5.8	2	to 12.0	%
Lymphocyte	17.6	19	to 53.0	%
Hemoglobin	12.4	11.5	to 15.0	g/dL
Platelets	43.4×10^4	15	to 35.0	10^4/L
CRP	0.57	0	to 0.30	mg/dL
RF	1.7	0	to 15.0	IU/ml
ACE	7.3	8.3	to 21.4	NU
sIL-2R	843	122	to 496	U/mL
IgG	1053	880	to 1800	mg/dL
IgM	142	52	to 270	mg/dL
ANA	<40		<40	times
SS-A	Negative			
SS-B	Negative			
PR3-ANCA	<1.0	0	to 3.4	U/ml
MPO-ANCA	<1.0	0	to 3.4	U/ml
PRP	Negative			
TpAb	Negative			

**Figure 2:**
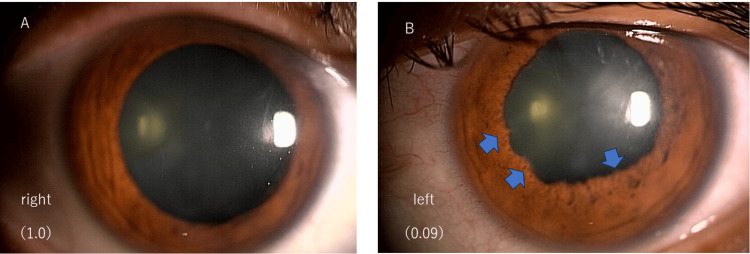
Anterior segment images at the initial examination (A) Right and (B) left. Inflammatory cells in the anterior chamber and keratic precipitate were observed in both eyes. Posterior iris synechiae were seen in the left eye (B: arrows). No IK was found in both eyes. IK: Interstitial keratitis

Ophthalmological examination revealed that her right CVA and left CVA were 1.0 and 0.09, respectively. Slit-lamp microscopy revealed inflammatory cells, posterior corneal deposits in both eyes, and posterior iris synechia in the left eye; however, IK was not observed (Figures 2A, 2B).

**Figure 3:**
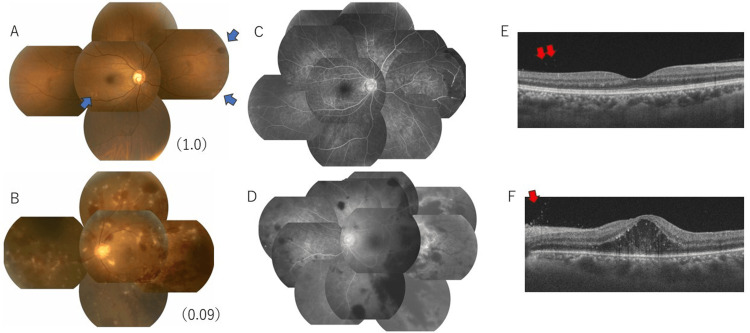
Multimodal fundus imaging at the initial visit (A, B) Panoramic fundus photograph. (A) The right eye showed retinal hemorrhage and vasculitis in the branches of the inferior temporal vein and most peripheral nasal area (blue arrows). (B) The left eye showed retinal vasculitis with papillary edema, extensive retinal hemorrhage, and cotton wool patches. (C, D) Fluorescein angiography. C) The retinal vasculitis and blockage due to hemorrhage were observed in the nasal retina. (D) The extensive arterial occlusion in the temporal retina. (E, F) OCT images. Vitreous cells were observed in both eyes (red arrows), and macular edema was observed in the left eye. A, C, E: right eye, B, D, F: left eye OCT: Optical coherence tomography

Gonioscopy revealed no nodules, peripheral anterior synechia, or angle hypopyons in either eye. Fundus examination revealed focal retinal hemorrhage and vasculitis in the right eye (Figure 3A) and extensive retinal vasculitis with papillary edema, extensive retinal hemorrhage, and multiple cotton wool patches in the left eye (Figure 3B). FA showed fluorescent leakage from both retinal arteries and veins, hemorrhagic blockage on the nasal side of the right eye (Figure 3C), and extensive arterial occlusion on the temporal retina of the left eye (Figure 3D). Optical coherence tomography revealed vitreous inflammatory cells in both eyes and macular edema in the left eye (Figures 3E, 3F).

**Figure 4:**
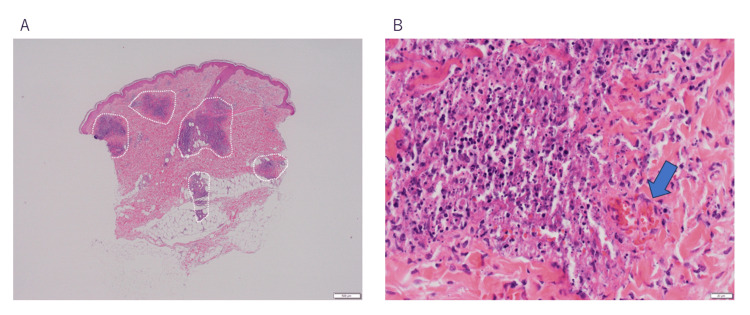
Dermatopathological findings of multiple erythema (A) Inflammatory cell infiltration was observed mainly around small blood vessels in the dermis to subcutaneous adipose tissue enclosed by a white dotted line. (B) High magnification of (A). Leukocytes, in which the nuclei were fractured, were infiltrated around the small vessels. Similar histology was seen in leukocytoclastic vasculitis. The arrow indicates capillary.

Audiometry revealed sensorineural hearing loss mainly in the high-frequency range on the right side and in the full-frequency range on the left side (Figure 1B). Histological examination of the erythematous lesions showed inflammatory cell infiltration, mainly around small blood vessels in the dermis to the subcutaneous adipose tissue (Figure 4A). At high magnification, it was observed that leukocytes with fractured nuclei infiltrated the small vessels, which is also seen in the histology of leukocytoclastic vasculitis (Figure 4B). The inflammatory cells were predominantly neutrophils and histiocytes with a small number of T lymphocytes. IgA, IgM, IgG, C1q, C3, C4, and fibrinogen levels were negative as seen using the direct fluorescent antibody technique (data not shown). There have been reports of CS with leukocytoclastic vasculitis, and the pathology of the erythema in this case was consistent with that seen in previous reports [7-11].

**Figure 5:**
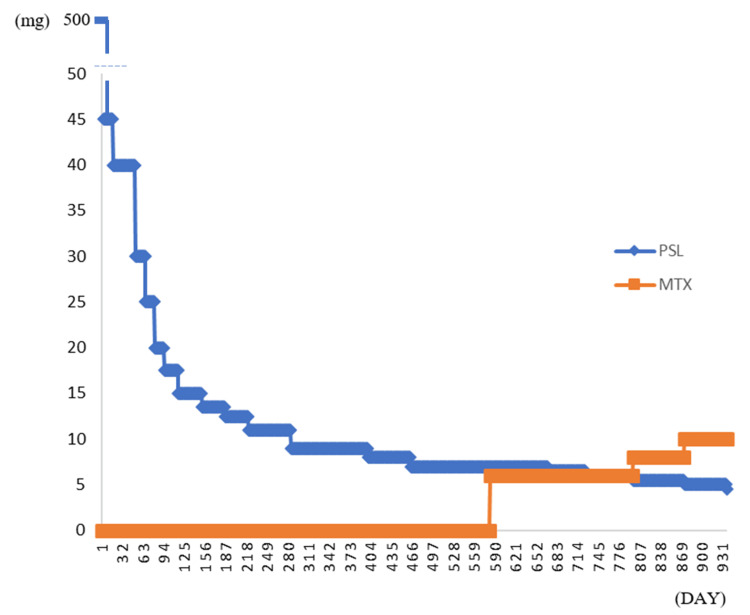
Course of administration of steroids and immunosuppressive drugs Steroid mini pulses were administered for three days and then tapered slowly. Methotrexate was started approximately 18 months after steroid administration. PSL: prednisolone; MTX: methotrexate

Chest radiography revealed no significant findings including bilateral hilar lymphadenopathy (BHL), and echocardiography revealed no abnormalities. Contrast-enhanced MRI and MRA of the patient’s head revealed no significant findings. Whole-body magnetic resonance imaging (diffusion-weighted whole-body imaging with background body signal; DWIBS) showed no obvious macro angiitis (data not shown). Based on the above findings, we considered this a case of atypical CS. As shown in Figure 5, the patient was treated with 500 mg methylprednisolone for three days, which was then switched to oral prednisolone and tapered very slowly.

**Figure 6:**
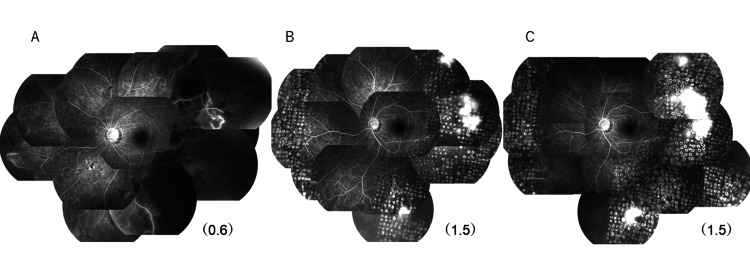
Follow-up fluorescein angiography images in the left eye (A) One month after the start of steroid administration. Extensive non-perfusion areas and fluorescent leakage from retinal vessels thought to be vasculitis were observed, but RNV was not detected. CVA: (0.6) (B) 1.5 years later, multiple RNV had grown in the area treated by photocoagulation. CVA: (1.5) (C) Multiple RNV had remained but had not increased at 2.5 years later. CVA: (1.5) RNV: Retinal neovascularization; CVA: corrected visual acuity of the decimal unit

Ophthalmologic treatments included mydriatic (tropicamide phenylephrine hydrochloride) three times per day for the left eye, steroids (betamethasone sodium phosphate) four times per day for both eyes and an antibiotic eye drop (levofloxacin hydrate) three times per day for both eyes. Mydriatic and antibiotic eye drops were stopped after one month, and steroid eye drop was stopped after six months. The skin erythema quickly resolved after steroid administration. Two weeks later, the macular edema disappeared, the CVA of the left eye improved to 0.6, and hearing tests also improved (Figure 1C). Retest of FA confirmed the non-perfused area (Figure 6A), so retinal photocoagulation was applied to the non-perfused area using a pattern-scanning laser device (MC500; NIDEK, Nagoya, Japan) to prevent retinal neovascularization (RNV). One and a half years later, because RNV occurred in the non-perfusion area where the laser was performed (Figure 6B), an additional laser was applied to the gap between the laser scars. At the last visit three years after the initial visit, the CVA was 1.5 in both eyes. RNV persisted but had not expanded, and vitreous hemorrhage did not occur (Figure 6C).

The patient continued prednisolone (4 mg) and methotrexate (10 mg) treatment with no recurrence of retinal vasculitis, multiple erythema, or inner ear symptoms (Figure 5).

## Discussion

The ocular manifestations of atypical CS include conjunctivitis, iritis, scleritis, and retinal arteriolar occlusion, which can be monocular or bilateral. The visual prognosis is relatively good, but hearing impairment is irreversible and responds poorly to treatment [4,12-14]. Early steroid administration is recommended when CS is diagnosed, and slow tapering of steroids is important to prevent irreversible hearing loss and serious life-threatening complications, such as aortitis [14,15]. It has also been reported that the mean time from ocular symptoms to the onset of inner ear symptoms in classic CS cases is two months, compared with 27.1 months in atypical CS [14,15]. Atypical CS is often difficult to diagnose, not only because of the long interval between the onset of ocular and inner ear symptoms but also because it is often associated with other collagen vascular disorders such as sarcoidosis, rheumatism, and Sjögren's syndrome. Our patient presented with inflammatory eye symptoms, including retinal vasculitis, skin symptoms, and high fever, but various autoantibodies and infectious tests were negative; therefore, we suspected atypical CS and performed a hearing test. A hearing test detected decreased hearing ability in both ears, which led to the diagnosis of atypical CS. As mentioned earlier, differentiation from other collagenous vascular diseases is often difficult, but in the present case, we made the differentiation, which was based on the following findings and laboratory data. Behçet's disease was ruled out because the patient had no history of oral or pubic ulceration, no hypopyon, and no diffuse fern-like fluorescent leakage on FA (Figures 3C, 3D). Sarcoidosis was also ruled out because chest radiography revealed no significant findings including BHL, although sIL2R was slightly elevated, ACE was within the normal limit in the lab data (Table 1) and histological examination of the erythematous lesions didn’t include non-caseating granuloma. As she had no joint symptoms and RF was within the normal limit (Table 1), rheumatoid arthritis was ruled out. She didn’t feel dry eye and mouth, and the titer of SS-A and SS-B antibodies were not elevated (Table 1), so Sjögren's syndrome was also ruled out. After steroid-based treatment, the ocular inflammation and hearing loss markedly improved. She was still taking 4 mg prednisolone and 10 mg methotrexate, which may have prevented recurrence of retinal vasculitis and hearing impairment during the three-year follow-up period. A pattern scanning laser (power: 370-400mW, size: 200µm spacing: 0.75, duration: 0.02sec) was used to treat the non-perfusion area to prevent RNV. However, 18 months later, RNV was observed within the area where the laser coagulation was performed in the left eye. Pattern-scanning lasers with short durations have been reported to have slightly weaker coagulation effects than conventional multicolor lasers [16]. After additional laser irradiation of the interstitial space between the coagulation spots, complete regression of the RNV was not achieved; however, there was no enlargement or vitreous hemorrhage. Considering this, conventional multicolor laser retinal coagulation or pattern-scanning laser coagulation with packed spacing may be preferable for non-perfusion areas associated with retinal vasculitis.

## Conclusions

Early diagnosis of CS is difficult because it is a rare disease that can be complicated by various autoimmune diseases, and there is a time lag between the onset of ocular inflammation and inner ear symptoms. However, early diagnosis and treatment are important, because delays in diagnosis can lead to irreversible hearing loss. For an early diagnosis, it is important to include CS in the differential diagnosis, and the search for related symptoms is the deciding factor in determining whether an early diagnosis can be made. We believe that this report provides information that will aid in the diagnosis of atypical CS. It should be noted that CS has been reported to have a high recurrence rate and therefore requires long-term follow-up, including collaboration with other medical departments.
